# Response to: Methodological and ethical considerations in evaluating centralized COVID-19 drug procurement: a letter to the editor

**DOI:** 10.3205/id000096

**Published:** 2025-11-07

**Authors:** Kathrin Marx, Sven Kalbitz, Nils Kellner, Maike Fedders, Christoph Lübbert

**Affiliations:** 1 Hospital Pharmacy, Hospital St. Georg, Leipzig, Germany; 2 Department of Infectious Diseases and Tropical Medicine, Hospital St. Georg, Leipzig, Germany; 3 Division of Infectious Diseases and Tropical Medicine, Department of Medicine I, Leipzig University Hospital, Leipzig, Germany; 4 Interdisciplinary Center for Infectious Diseases, Leipzig University Hospital, Leipzig, Germany

## Letter to the editor

Dear colleagues,

We have read your letter to the editor with interest [1]. The authors of the letter believe that a broader discussion of methodological and ethical considerations is needed to fully inform future healthcare planning and drug policy.

As already described in the introduction to our publication, a temporal study of the hospitalization and treatment of COVID-19 patients with the drugs centrally procured by the Federal Ministry of Health (Bundesministerium für Gesundheit, BMG) was conducted at the St. Georg Hospital in Leipzig, Germany. The focus was on the temporal relationship between the provision and use of medicines in the context of the recommendations of the European Medicines Agency (EMA) and the resulting recommendations of the professional medical societies. The aim of this analysis was to describe and classify the epidemiological development of hospital stays in the temporal context of the drugs procured centrally by the Federal Ministry of Health, the recommendations of the EMA and the professional societies on the targeted use and clinical implementation of the available treatment options. St. Georg Hospital Leipzig is a specialist and maximum care hospital with its own large department for infectious diseases and tropical medicine, which acts as an infectious disease competence center for the Free State of Saxony. COVID-19 patients were primarily treated on the two infectiology wards with a total of 44 beds and special isolation conditions. For a comparable control group, as proposed by the authors of the letter, a hospital with comparable structures would be required (planned beds in the Department of Infectious Diseases and Tropical Medicine and a designated supra-regional infectiology center). In a control group, the comparability of standard treatment procedures, the experience of clinicians and the advisory role of specialist infectious disease physicians must be ensured.

During the study period, the course of the SARS-CoV-2 pandemic was divided into six epidemiological phases or waves. Several SARS-CoV-2 variants were classified as variants of concern (VOCs). In particular, the proportion of VOCs in circulation was used to define the waves. Given the dynamics of the pandemic described here, propensity score matching is not considered a sufficient option for balancing the treatment groups and controlling for confounding factors in the baseline situation. Statistical adjustments for potential confounders are not limited to age, gender and comorbidities; factors such as treatment during a particular pandemic wave or the presence of VOC must also be taken into account. As our municipal hospital does not have a dedicated department for epidemiology and medical statistics and we are primarily clinicians, we limited our analysis to the descriptive level. We deliberately opted for a descriptive analysis and did not perform multivariate adjustment for important confounders such as comorbidities, age and immunization status, which limits the interpretability of the study. However, our study was not primarily designed to analyze treatment outcomes in SARS-CoV-2 patients.

In our temporal analysis of the use of centrally procured medications to treat patients with SARS-CoV-2 infection, we did not have access to information on the extent of outpatient care for these patients, nor on treatment outcomes or potential confounders in the evaluation of outpatients. In addition, we ask the authors of the letter to the editor to consider that this temporal analysis was submitted on May 8, 2023, and included cases up to February 28, 2023. At the time of publication, we did not have access to information on the efficiency or equity of centralized versus decentralized procurement approaches in other countries.

Our study is only one part of a holistic approach to maximize preparedness and ensure that policies are implemented with optimal effectiveness in the event of future health crises. We support the authors’ recommendation to adopt a multidisciplinary approach in subsequent studies that combines causal inference and ambulatory information to generate actionable public health insights.

## Notes

### Competing interests

The authors declare that they have no competing interests.
